# Effect of Composition Adjustment on the Thermoelectric Properties of Mg_3_Bi_2_-Based Thermoelectric Materials

**DOI:** 10.3390/mi14101844

**Published:** 2023-09-27

**Authors:** Jianbao Yang, Zhibin Wang, Hong Zhao, Xinyu Luo, Wenyuan Han, Hao Wang, Linghao Meng, Xinqi She, Anlong Quan, Yixin Peng, Guoji Cai, Yi Liu, Yong Tang, Bo Feng

**Affiliations:** 1 Institute of Engineering and Technology, Hubei University of Science and Technology, Xianning 437100, China17787474499@163.com (H.Z.); 17307256568@163.com (X.L.); 17710522492@163.com (W.H.); 15271899946@163.com (Y.P.); 2 School of Mechanical and Electrical Engineering, Wuhan Donghu University, Wuhan 430070, China; whjiaguoji@163.com (G.C.); tangyong_tt@163.com (Y.T.); 3 The State Key Laboratory of Refractories and Metallurgy, Wuhan University of Science and Technology, Wuhan 430081, China

**Keywords:** Mg_3_Bi_2_, composition adjustment, acceptor effect, thermoelectric properties

## Abstract

Thermoelectric materials are widely used in refrigeration chips, thermal power generation, catalysis and other fields. Mg_3_Bi_2_-based thermoelectric material is one of the most promising thermoelectric materials. Herein, the Mg_3_Bi_2_-based samples were prepared by high temperature synthesis, and the influence of Mg/Sb content on the electrical transport properties and semi-conductivity/semi-metallicity of the materials has been studied. The results indicate that the efficiency of introducing electrons from excess Mg prepared by high temperature synthesis is lower than that introduced by ball milling, due to the high vapor pressure of Mg. The doping of Sb/Te at the Bi site would make it easier for the material to change from p-type conduction to n-type conduction. With the increase in Mg content, the semi-conductivity of the material becomes weaker, the semi-metallicity becomes stronger, and the corresponding conductivity increases. With the increase in Sb content, the samples exhibit the opposite changes. The highest power factor of ~1.98 mWm^−1^K^−2^ is obtained from the Mg_3.55_Bi_1.27_Sb_0.7_Te_0.03_ sample.

## 1. Introduction

Thermoelectric materials can realize the mutual conversion of thermal energy and electrical energy and can reduce the work function and improve the catalytic efficiency of chemical reactions [1,2,3]. The conversion efficiency of thermoelectric materials is evaluated by dimensionless thermoelectric figures of merit (*ZT* = *S*^2^*σT*/*κ*, *S*, *σ*, *T*, *κ* is the Seebeck coefficient, the electrical conductivity, the absolute temperature, the thermal conductivity, respectively) [4,5]. In order to achieve high thermoelectric conversion efficiency, thermoelectric materials are supposed to exhibit a high power factor (*PF* = *S*^2^*σ*) and low thermal conductivity, which is “phonon-glass and electron-crystal” [6,7]. In accordance with this principle, the representative materials of Bi_2_Te_3_-based [8,9], PbTe-based [10,11] and SiGe-based [12,13] thermoelectric materials have been developed in the past several years, and have been used in refrigeration [14,15], waste-heat power generation [16,17], micro-electronic devices [18,19], etc. However, traditional thermoelectric materials have the problems of poisonous elements [20,21], high costs [22] and so on, which have limited their further applications.

The new type of Mg_3_Bi_2_-based thermoelectric material is low-cost, non-toxic, and displays excellent thermoelectric performances, which make it one of the most promising thermoelectric materials for large-scale commercial application [23,24,25,26,27]. Mg_3_Bi_2_ (*P*-3*m*1 space group)-based thermoelectric material exhibits a special layered structure, with the Mg^+^ layer and the (Mg_2_Bi_2_)^−^ layer alternately stacked along the *c* axis [28,29]. The binding force between the Mg^+^ layer and the (Mg_2_Bi_2_)^−^ layer is relatively weak, and Mg vacancies are easy to generate in the material, exhibiting intrinsic p-type conduction [30]. Through composition adjustment, such as Te doping, a donor effect can be introduced with an increase in the number of electrons, and the material can be adjusted to n-type conduction. The thermoelectric temperature difference realized by the devices made of n-type Mg_3_Bi_2_-based thermoelectric material is significantly higher than that of commercial n-type Bi_2_Te_3_-based thermoelectric devices at the same temperature.

Mg-Bi-Sb-based thermoelectric materials are the most promising new materials to replace commercial bismuth telluride thermoelectric materials. They have great advantages in thermoelectric performance, maximum cooling temperature difference and cost. It is difficult for high-energy ball milling to meet the requirements of mass industrial production, but the smelting method is easy to realize. Previous literature tends to use the ball milling method to prepare this kind of material [31], but there are some problems, such as non-repeatability and non-uniformity in large-scale industrial application [32]. Herein, a high-temperature reaction method was adopted, which is easy to realize for large-scale industrial application [33]. Based on prepared Mg_3_Bi_2_-based thermoelectric materials, a composition adjustment was adopted to turn the material to n-type conduction and improve the power factor by Mg excess and Sb/Te doping [1].

## 2. Experimental Section

### 2.1. Materials and Synthesis

Samples with the chemical composition of Mg_3_Bi_2_-based thermoelectric materials were synthesized by a high-temperature reaction method combined with resistance pressing sintering (RPS) [34,35]. The commercial Mg (99.99%), Bi (99.999%), Te (99.999%) and Sb (99.999%) were used as starting materials. The raw materials come from China National Pharmaceutical Group. The powder, weighed according to the stoichiometric ratio and mixed in a glove box, was put into a capped graphite mold, then put into a quartz tube filled with argon and annealed at 1173 K for two hours. The corresponding heating rate is 50 K/min, and the cooling method is furnace cooling. The obtained blocks were crushed into powder in a glove box and then sintered by resistance pressing sintering (RPS) under the axial compressive pressure of 50 MPa under an argon atmosphere at 873 K for 10 min. Machine demolding was used to avoid sample breakage. The inner cavity size of the graphite mold is Φ 20 × 13 mm. The heating rate of the sintering process is 50 K/min, the cooling rate is 50 K/min, the pressurization method is to evenly increase the pressure of 50 MPa within 12 min, and the pressure relief method is to evenly release the pressure within 12 min. After sintering to obtain the block, the graphite paper on the surface of the sample was ground off with sandpaper, and then the sample of the required size for testing was cut using wire cutting. Before testing, the sample would be washed clean with water and alcohol, and dried in a vacuum atmosphere in a drying oven for more than 48 h.

### 2.2. Measurements

The X-ray diffraction (XRD) patterns were collected with the PANalytical X’Pert Pro (Malvern, UK) diffractometer for phase identification. The microstructures of the samples were observed using field emission scanning electron microscopy (SEM, FEI, Nova 400 Nano, FEI, Eindhoven, The Netherlands) and the chemical compositions were analyzed using energy-dispersive X-ray spectroscopy (EDS). In order to observe the morphology of naturally formed grains, the fracture morphology of naturally broken samples was observed. For the measurement of electrical transport properties, the sintered bulks were cut into bar-shaped specimens with the dimensions of 3 mm × 3 mm × 15 mm along the radial direction. The electrical resistivity (*ρ*) and Seebeck coefficient (*S*) were measured by commercial equipment (ZEM-3, ADVANCE-RIKO, Yokohama, Japan) with an uncertainty of ~5%. For the measurement of the band gap between the valence band and the conduction band, the infrared spectroscopy was obtained by a Fourier transform infrared/Raman spectrometer (VERTEX 70, Bruker, Mannheim, Germany).

## 3. Results and Discussion

**Figure 1:**
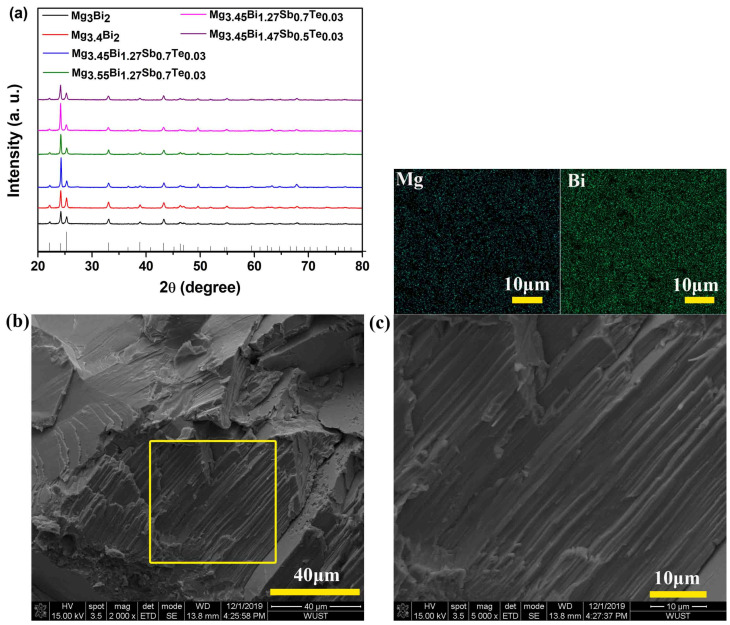
(**a**) Room temperature powder XRD patterns for Mg_3_Bi_2_-based series of samples; (**b**) SEM image of Mg_3.4_Bi_2_; (**c**) the enlarged view of the frame selected area in (**b**) and corresponding EDS mappings of Bi and Mg.

It can be seen from Figure 1a that the diffraction peaks of the Mg_3_Bi_2_-based series of samples prepared by high-temperature reactions correspond to the standard cards one by one, without any obviously differing peaks. The results indicate that smelting, combined with spark plasma sintering, is an effective method to prepare Mg-Bi-Sb-based thermoelectric materials. This method can promote the industrialization of Mg-Bi-Sb-based thermoelectric materials. Figure 1b shows the SEM pictures of the fracture morphology of the Mg_3.4_Bi_2_ sample. It can be seen that the grains of the sample exhibit a lamellar structure, similar to Bi_2_Te_3_. The EDS results show that the elements are evenly distributed without obvious component segregation.

**Figure 2:**
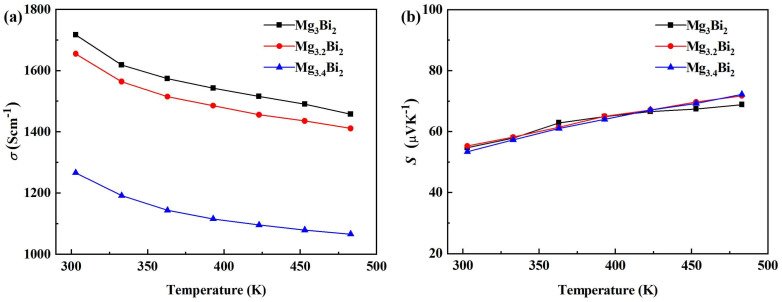
The electrical conductivity (*σ*) (**a**) and the Seebeck coefficient (*S*) (**b**) varying with temperature for Mg_3+*x*_Bi_2_ (*x* = 0, 0.2, 0.4) samples.

**Figure 3:**
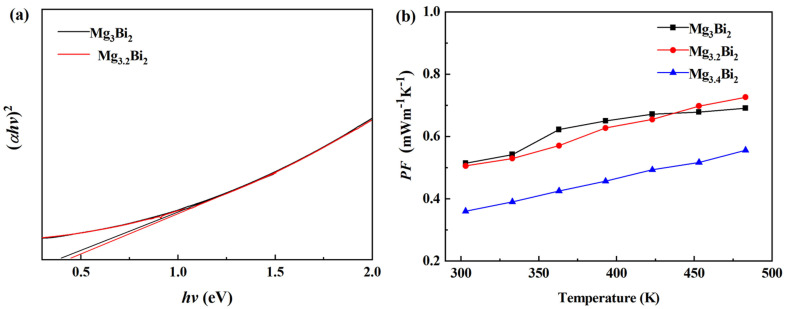
The diagram of band gap calculation by extrapolation (**a**) and power factor (*PF*); (**b**) varying with temperature for Mg_3+*x*_Bi_2_ (*x* = 0, 0.2, 0.4) samples.

The changed pattern of electrical conductivity (*σ*) varying with temperature of the Mg_3+*x*_Bi_2_ (***x*** = 0, 0.2, 0.4) samples is shown in Figure 2a. It can be seen that the electrical conductivity of all samples decreased with the increase in temperature. The electrical conductivity of Mg_3_Bi_2_ at room temperature was ~1720 S cm^−1^. With the increase in Mg content (***x***), the electrical conductivity decreased in the testing temperature range. In order to analyze the change in electrical properties, we tested the infrared spectrum of the samples and calculated the band gap by extrapolation as shown in Figure 3a [36,37]. With the increase in Mg content, the extrapolation curve exhibits a downward trend, and the corresponding band gap shows an upward trend. The wider the band gap, the more difficult it is for the carriers to jump over the forbidden band, which leads to a decrease in carrier concentration and electrical conductivity [38,39]. On the one hand, excess Mg enters vacancies, weakening the acceptor effect, which could reduce the number of hole carriers [40]. On the other hand, according to the band gap testing results, the widening of the band gap results in a decrease in the number of current carriers passing through the band gap, which also reduces the number of hole carriers. The corresponding modifications have been added to the article.

The Seebeck coefficient (*S*) of Mg_3+*x*_Bi_2_ (*x* = 0, 0.2, 0.4) varying with temperature is shown in Figure 2b. It can be seen that the Seebeck coefficients of the samples are positive, exhibiting p-type conduction. This is related to the high vapor pressure of Mg, which results in easily-formed Mg vacancies during the process of high-temperature preparation [41]. Mg vacancies can enhance the acceptor effect and increase the hole concentration, so that most carriers in the material are holes [42,43]. With the increase in Mg content, the change in the Seebeck coefficient is not obvious. As shown in Equation (1):

(1) $$S=\pm \frac{k_{B}}{e}\left[\gamma +2+\ln\frac{2{\left(2\pi m*k_{B}T\right)}^{3/2}}{h^{3}n}\right]$$

The Seebeck coefficient is mainly determined by the influence of the effective mass (m*) and carrier concentration (*n*). On the one hand, the Seebeck coefficient is related to the parameters regarding the carrier concentration (related to electrical conductivity), and on the other hand, it is also related to effective mass. Due to the increase in Mg, the electrical conductivity and the number of hole carriers decrease, possibly due to a decrease in effective mass, resulting in a small change in the Seebeck coefficient. The power factor (*PF*) of all Mg_3+*x*_Bi_2_ (*x* = 0, 0.2, 0.4) samples increased with the increase in temperature, and the maximum value of ~0.73 mWm^−1^K^−2^ was obtained at 483 K.

For the ball milling method, when the excess content of Mg reached ~6.7% (0.2/3, corresponding to Mg_3.2_Bi_2_), the Seebeck coefficient adjusted to a negative value [1]. For the high temperature reaction method, due to the high temperature of the process, the Mg volatilized easily in the high vapor pressure, and the efficiency of introducing the electrons through Mg decreased, according to defect chemistry theory [44,45]. When the excess content of Mg reached ~13. 3% (0.4/3, corresponding to Mg_3.4_Bi_2_), the Seebeck coefficient was still positive. Therefore, we adopted 35% Sb and 1.5% Te to dope at Bi sites to introduce more electrons, followed by optimizing Mg content to adjust the half-metallicity/semi-conductivity and electrical properties of the material [1].

**Figure 4:**
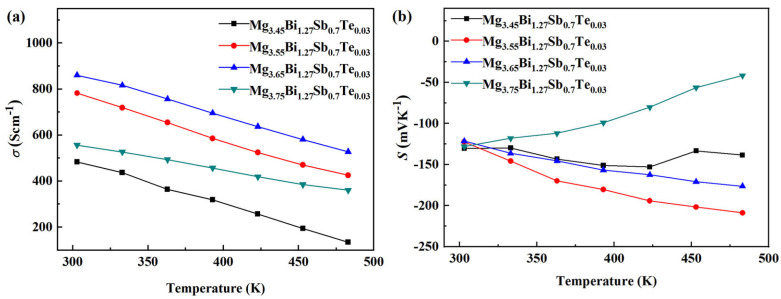
The electrical conductivity (*σ*) (**a**) and Seebeck coefficient (*S*) (**b**) varying with temperature for Mg_3.45+*x*_Bi_1.27_Sb_0.7_Te_0.03_ (*x* = 0, 0.1, 0.2, 0.3) samples.

**Figure 5:**
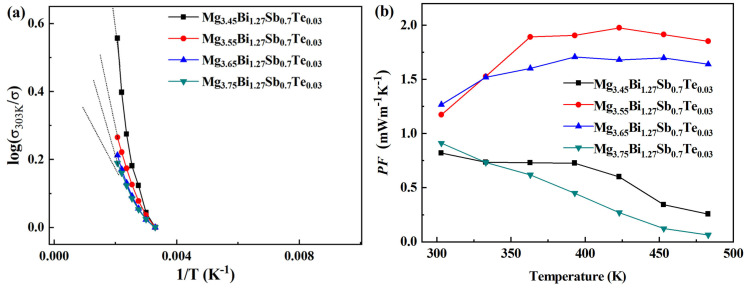
The temperature dependence of electrical conductivity (*σ*) (**a**) and power factor (*PF*) (**b**) varying with temperature for Mg_3.45+*x*_Bi_1.27_Sb_0.7_Te_0.03_ (*x* = 0, 0.1, 0.2, 0.3) samples.

The electrical conductivity (*σ*) varying with temperature for the Mg_3.45+*x*_Bi_1.27_Sb_0.7_Te_0.03_ (*x* = 0, 0.1, 0.2, 0.3) samples is shown in Figure 4. It can be seen that, with the increase in *x* from 0 to 0.2, the electrical conductivity increases in the testing temperature range. When *x* increases from 0.2 to 0.3, the corresponding electrical conductivity decreases, but *x* is still higher than zero. Through the log conversion of the relationship between the electrical conductivity data and the temperature (*σ*_303K/_*σ*)~(1/T), as shown in Figure 5a, it can be seen that with the increase inf Mg content, the slope of the curve decreases, the rake ratio decreases, the half-metallicity of the material increases and the semi-conductivity decreases [1].

Figure 4b shows the Seebeck coefficient (*S*) varying with temperature for the Mg_3.45+*x*_Bi_1.27_Sb_0.7_Te_0.03_ (*x* = 0, 0.1, 0.2, 0.3) samples. The Seebeck coefficients of all samples are negative in the whole temperature range, exhibiting n-type conduction [46]. When *x* increases from 0 to 0.1, the absolute value of the Seebeck coefficient shows an upward trend, with the maximum value being ~−210 μVK^−1^ at 483 K. When *x* increases from 0.1 to 0.3, the absolute value of the Seebeck coefficient decreases. The power factor (*PF*) varying with temperature for the Mg_3.45+*x*_Bi_1.27_Sb_0.7_Te_0.03_ (*x* = 0, 0.1, 0.2, 0.3) samples is shown in Figure 5b. The highest power factor at room temperature is ~1.27 mWm^−1^K^−2^, obtained from Mg_3.65_Bi_1.27_Sb_0.7_Te_0.03_. The highest power factor in the whole temperature range was ~1.98 mWm^−1^K^−2^ at 423 K, obtained from Mg_3.55_Bi_1.27_Sb_0.7_Te_0.03_. The actual composition of the highest Mg content of the sample (3.75) as detected by EDS was ~3.69. Compared to doping experiments with materials such as bismuth telluride, this deviation is significant due to the volatility of Mg. The detection results of the XRD equipment can indirectly reflect that there is no excess of the Mg element. Adding more Mg has two considerations. On the one hand, Mg is easily volatile and may be lost during high-temperature preparation. On the other hand, our goal is to make n-type materials, and we need to reduce the number of hole carriers. Adding more magnesium is beneficial for suppressing the acceptor effect, aimed at adjusting the material conductivity to n-type.

**Figure 6:**
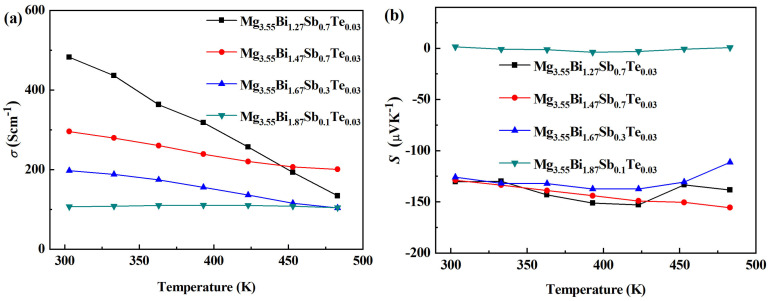
The electrical conductivity (*σ*) (**a**) and Seebeck coefficient (*S*) (**b**) varying with temperature for Mg_3.55_Bi_(1.27+*x*)_Sb_(0.7−*x*)_Te_0.03_ (*x* = 0, 0.2, 0.4, 0.6) samples.

**Figure 7:**
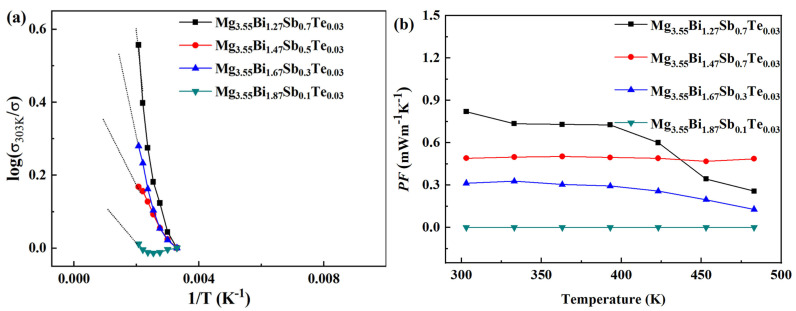
The temperature dependence of electrical conductivity (*σ*) (**a**), and power factor (*PF*) (**b**) varying with temperature for Mg_3.55_Bi_(1.27+*x*)_Sb_(0.7−*x*)_Te_0.03_ (*x* = 0, 0.2, 0.4, 0.6) samples.

The electrical conductivity (*σ*) varying with temperature for the Mg_3.55_Bi_(1.27+*x*)_Sb_(0.7−*x*)_Te_0.03_ (*x* = 0, 0.2, 0.4, 0.6) samples is shown in Figure 6. The electrical conductivity of all samples decreased with the increase in temperature, indicating semiconductor-transporting behavior. With the increase in Sb content, the electrical conductivity increased in most temperature ranges. The relation of log (*σ*_303K/_*σ*)~(1/T), as shown in Figure 7a, means that with the increase in Sb content, the slope of the curve becomes steeper, corresponding to the semi-conductivity of the materials increasing, while the semi-metallicity weakens [1].

Figure 6b shows the Seebeck coefficient (*S*) varying with temperature for the Mg_3.55_Bi_(1.27+*x*)_ Sb_(0.7−*x*)_Te_0.03_ (*x* = 0, 0.2, 0.4, 0.6) samples. When the Sb content is 0.1 (*x* = 0.6), the Seebeck coefficient of the sample is very small. When the Sb content is 0.3, 0.5 or 0.7 (*x* = 0.4, 0.2, 0), the absolute values of the Seebeck coefficient from ~303 K to ~350 K vary a little. In the relatively high temperature range, when the Sb content is 0.5 (*x* = 0.2), the absolute value of the Seebeck coefficient is higher, with the highest value of −156 μVK^−1^ being obtained at 483 K. In general, as the Sb content increases from 0.1 to 0.7 (from *x* = 0.6 to *x* = 0.1), the power factor shows an upward trend, with the highest power factor in the whole temperature range being ~0.82 mWm^−1^K^−2^ at 303 K, obtained from Mg_3.55_Bi_1.27_Sb_0.7_Te_0.03_.

## 4. Conclusions

Herein, Mg_3_Bi_2_-based thermoelectric materials were prepared by high temperature reaction. The effects of composition adjustment on their electrical transporting properties have been studied. The results indicate that the electrical conductivity decreased when the content of Mg increased, due to the filling of Mg vacancies and the expansion of the band gap, while the Seebeck coefficient does not change significantly. During the high temperature process, the efficiency of introducing electrons is lower than that of ball milling, due to the high vapor pressure of Mg. After doping Sb and Te at Bi sites, the material shows n-type conduction. On this basis, the effects of the content of Mg/Sb have been studied. It is found that with an increase in Mg content, the semi-conductivity of the material becomes weak, the semi-metallicity becomes strong, and the corresponding conductivity increases. With an increase in Sb content, the sample shows the opposite change. The highest power factor obtained from the Mg_3.55_Bi_1.27_Sb_0.7_Te_0.03_ sample is ~1.98 mWm^−1^K^−2^.

## Data Availability

The raw/processed data required to reproduce these findings cannot be shared at this time as the data also forms part of an ongoing study.
